# Lightweight design and post-impact attitude stability of extra-high grade guardrail based on 700L high-strength steel

**DOI:** 10.1371/journal.pone.0358588

**Published:** 2026-09-15

**Authors:** Kunmiao Xu, Shuai Gong, Wei Wang, Xin Wang, Siyuan Liu, Shuming Yan

**Affiliations:** 1 Shandong Hi-Speed Company Limited, Jinan, China; 2 Beijing Hualuan Traffic Technology Co., Ltd., Beijing, China; Swiss Federal Technology Institute of Lausanne, SWITZERLAND

## Abstract

To address the issues of excessive self-weight and insufficient post-impact attitude stability inherent in traditional HA-level (extra-high grade) beam-post guardrails due to oversized cross-sections, this paper proposes a lightweight design method utilizing 700L high-strength alloy steel. Through topology optimization, a novel guardrail structure comprising “inclined H-shaped posts” and “rectangular tube beams” was developed. Full-scale vehicle crash tests show that under the HA-level heavy-vehicle impact matrix specified in JTG B05-01–2013—namely a 25 t extra-large passenger bus, a 40 t heavy truck, and a 55 t tractor-semitrailer—the guardrail’s containment and redirection functions meet the corresponding HA-level heavy-vehicle requirements. None of the three vehicles rolled over, overrode, or straddled the guardrail, and all exited with stable post-impact attitude. Vehicle lateral intrusion is substantially reduced: under the 55 t tractor-semitrailer collision condition, the maximum dynamic equivalent vehicle lateral intrusion value (VIn) is reduced by 64.2% compared with conventional guardrails. In terms of lightweight design, the weight per linear meter of the guardrail is decreased from 230 kg to 180 kg, representing a reduction of 21.7%. Economic analysis shows that the reduction in material consumption lowers the overall material cost by approximately 10%–15%. This study demonstrates a material-structure co-design method that simultaneously improves safety, lateral intrusion resistance, lightweight level and economy for HA-level heavy-vehicle conditions, providing a theoretical basis and technical support for the engineering application of a new generation of high-performance bridge guardrails.

## 1. Introduction

In recent years, the construction scale of long-span cable-supported bridges (e.g., cable-stayed and suspension bridges) in China has continued to expand. Such bridges are sensitive to dead load variations, and key load-bearing components like stay cables and hangers are often installed adjacent to traffic lanes. Collisions with out-of-control vehicles can easily cause local structural damage or even catastrophic consequences [1–3]. As critical safety facilities on bridge decks, bridge guardrails must not only possess a sufficient protection level to effectively contain and redirect errant vehicles but also strictly control the dynamic vehicle ingress during collisions to prevent impacts with rearward cables and other key components [4].

The beam-post steel guardrail has become one of the preferred forms of guardrails for long-span cable bridges due to its good permeability, moderate self-weight, and convenient construction. However, the currently widely used Q355 steel guardrails often rely on increasing the sectional dimensions and thicknesses of components to improve stiffness and strength so as to achieve high protection levels and excellent anti-rollover performance. This results in a significant increase in self-weight and steel consumption, which not only contradicts the requirements of lightweight bridge design but also leads to problems such as increased project cost, low material utilization efficiency, and insufficient environmental performance [5,6]. A deeper contradiction lies in that traditional empirical design methods can hardly balance lightweight design and high performance (especially anti-rollover capability) in the vehicle-guardrail collision dynamic behavior, making it difficult to realize multi-objective collaborative optimization.

Existing studies mostly focus on a single dimension, either focusing on material replacement with higher-strength steel or committing to empirical improvement of local components [7–14]. Generally, there is a lack of a systematic perspective of “material-structure” integrated collaborative design, and the advanced material performance and cutting-edge structural optimization methods have not been effectively integrated. Recent studies have begun to explore the application of high-strength steels or optimization techniques in roadside safety hardware. Wei et al. [15] proposed a lightweight corrugated beam guardrail using HR700F high-strength steel, achieving a 44% weight reduction through cross-sectional optimization and full-scale crash test validation. Zhang and Bu [16] demonstrated the successful application of B750HL high-strength steel for bridge barrier components, confirming its protective capacity through both finite element simulations and full-scale-vehicle crash tests. Soekardjo et al. [17] quantified that the use of high-grade steel could reduce total reinforcing bar weight in road barrier design and cut costs by approximately 26%. Ozcanan and Atahan [13] applied surrogate-based optimization to improve the crash performance of W-beam guardrails according to EN 1317 standards. However, a critical gap remains in the material-structure co-design approach for extra-high grade guardrails, particularly in the systematic integration of advanced material characterization, topology optimization, and multi-objective collaborative optimization verified through rigorous full-scale testing.

Therefore, a design method integrating materials science and structural optimization is proposed. By integrating the mechanical performance advantages of 700L high-strength alloy steel and the configuration potential of topology optimization, the technical bottlenecks of high-protection grade guardrails in “lightweighting” and “anti-rollover” are broken through. This study realizes material thinning through the application of high-strength steel and achieves “on-demand material distribution” through topology optimization. Systematic demonstration is carried out from the aspects of collaborative design method, anti-rollover mechanism, lightweight effect and economy, providing theoretical support and engineering reference for the research and development of a new generation of high-performance and lightweight bridge guardrails.

## 2. Material and structural collaborative design method

### 2.1. Selection and performance characterization of high-strength steel materials

The conventional Q355 steel widely used in beam-post guardrails can no longer meet the comprehensive demands for “high strength, lightweight, and high toughness” materials under high protection levels. Therefore, this study employs 700L high-strength low-alloy steel. This material achieves a synergy of fine-grain strengthening and precipitation strengthening through micro-alloying and controlled rolling and cooling processes, offering both high strength and good plasticity.

Room temperature tensile tests were carried out on 3 ~ 6 mm thick 700L steel plates in accordance with the Chinese national standard GB/T 228.1–2021 (Metallic materials—Tensile testing—Part 1: Method of test at room temperature), and the results were compared with Q355 steel as shown in Table 1. Note that the 700L values represent measured properties from the actual material used, while the Q355 values represent the minimum requirements specified in the Chinese national standard GB/T 1591–2018.

**Table 1:** Comparison of mechanical properties between 700L and q355 alloy steels.

Performance Parameter	700L (Measured)	Q355 (Standard Requirement)	Performance Improvement
Lower Yield Strength (MPa)	626-720	≥355	76-102%
Tensile Strength (MPa)	704−795	470-630	49-69%
Elongation after Fracture (%)	20.5−27.0	≥21	Equivalent
Strength-Plasticity Product (GPa·%)	14.4−21.4	9.9-13.2	45-62%

Analysis of the above table data shows that:

(1) The yield strength of 700L is about 1.7 ~ 2.0 times that of the Q355 standard minimum. According to the strength criterion, when bearing the same load, the design thickness of the main components can be reduced accordingly, providing a direct basis for “high-strength thinning.”
(2) While maintaining high strength, the elongation after fracture of 700L is not less than 20.5%, and its plasticity is comparable to that of Q355, ensuring that the components still maintain structural integrity under large deformation during collision and avoid brittle fracture.
(3) The strength-plasticity product is a comprehensive index to evaluate the collision energy absorption capacity of materials. The strength-plasticity product of 700L is higher than that of Q355, indicating that it can absorb more energy per unit volume during collision, providing better buffering performance for the guardrail system.

The above results indicate that while 700L high-strength steel achieves “high strength and thickness reduction,” its excellent ductility ensures structural integrity during collisions. More importantly, its enhanced strength-ductility product (14.4–21.4 GPa·%) means the material can absorb more energy during deformation. This directly translates to outstanding crash-energy absorption potential for guardrail components such as posts and beams: under high-speed impact, the components can more effectively dissipate the vehicle's kinetic energy through large plastic deformation, thereby providing stronger buffering capacity for the entire guardrail system. This serves as a key material foundation for improving the crashworthiness of HA-level heavy-vehicle guardrails. Regarding hot-dip galvanizing for corrosion protection, 700L high-strength steel follows standard galvanizing procedures similar to Q355; no special process is required, though typical precautions for high-strength steels (such as controlled cooling after galvanizing to avoid hydrogen embrittlement) should be observed.

### 2.2. Structural design based on topology optimization

#### 2.2.1. Establishment of optimization model.

Using the initial envelope space of the guardrail posts and rails as the design domain, topology optimization was performed employing the variable density method based on the Solid Isotropic Material with Penalization (SIMP) interpolation model. This method transforms the discrete material distribution problem into a continuous optimization problem with element relative density as the design variable. The finite element model for topology optimization was established using 8-node hexahedral solid elements with an average mesh size of 10 mm, resulting in approximately 120,000 elements for the post design domain and 80,000 elements for the beam design domain. The SIMP penalization factor was set to p = 3, which is the standard value for structural topology optimization to promote clear 0–1 material distribution. A mesh independence filter with a radius of 1.5 times the element size was applied to eliminate checkerboard patterns and ensure mesh-independent solutions.

According to the HA-level crash conditions specified in the Highway Guardrail Safety Performance Evaluation Standard (JTG B05-01–2013, Clause 6.2.2, Table 6.2.2−1: 25-ton bus at 85 km/h, 20° impact angle), the impact was simplified into a static concentrated load P applied at the corresponding height on the beam via the energy equivalence principle. The equivalent static load P was calculated such that the external work done by P equals the lateral component of the vehicle kinetic energy (863 kJ for the bus case). This load case was selected for optimization because the bus impact represents the most critical loading condition for the upper portion of the guardrail where rollover initiation occurs; the subsequent full-scale crash tests with higher-energy vehicles (40 t truck and 55 t tractor-semitrailer) serve as validation of the optimized design under more severe conditions.

This quasi-static simplification is considered a reasonable approximation for deriving the primary load path in guardrail topology optimization for the following reasons. First, the dominant failure mode of a beam-column guardrail under a heavy-vehicle side impact is global bending and plastic hinge formation, a process primarily governed by the system's plastic limit load and energy dissipation capacity rather than high-frequency vibration modes. Second, the principle of energy equivalence, which equates the external work done by the pseudo-static load to the vehicle's initial kinetic energy, has been widely adopted in impact-resistant structural design to capture the critical load transfer path efficiently. Third, previous studies on similar vehicle-barrier systems demonstrated that the optimal topology from quasi-static optimization yields excellent performance under subsequent dynamic crash test validation [18,19].

Justification for the absence of an explicit dynamic finite element validation in the present study. An explicit dynamic finite element model of the final as-built guardrail is not included in this work. This is justified on two grounds. First, the present study is primarily an experimental verification study: the optimized configuration was directly evaluated through three full-scale crash tests, which are the highest level of evidence recognized by JTG B05-01–2013 and provide direct, standard-compliant measurements of the acceptance quantities D, W, VI, and VIn. Second, the role of topology optimization in this study is to generate a candidate load path rather than to predict final crash responses; the subsequent engineering interpretation and full-scale physical tests serve as the validation step. Nevertheless, a dynamic FE validation run of the final H-post/rectangular-tube guardrail under the 55 t tractor-semitrailer condition is planned to provide a numerical link between the optimization and the tests and will be reported in a follow-up publication.

Optimization model summary:

Objective: Minimize structural compliance C to maximize global stiffness.

Constraint: A volume fraction Vf ≤ 0.4.

Design variable: Element relative density ρe (0 ≤ ρe ≤ 1).

Load case: A static equivalent load P = 285 kN derived from the HA-level bus impact energy, applied at the rail height of 1.2 m above the deck.

Design domain dimensions: Post domain: 0.3 m wide × 1.58 m tall × 0.15 m deep; Beam domain: 0.2 m tall × 4.0 m long (two post spacings) × 0.12 m deep. By solving this optimization problem, the optimal distribution of materials in the design domain is sought, so as to obtain an efficient stress configuration with clear force transmission paths. It should be noted that while the optimizer maximizes global stiffness (minimizes compliance), the volume fraction constraint of 0.4 naturally removes low-stressed material primarily from the upper portion of the post where bending moments are smaller. This results in a tapered section that is relatively more flexible at the top—a beneficial byproduct for anti-rollover performance that was recognized and deliberately preserved during the engineering interpretation phase, rather than being an explicit objective of the optimization itself.

#### 2.2.2. Optimization results and configuration evolution.

**Fig 1:**
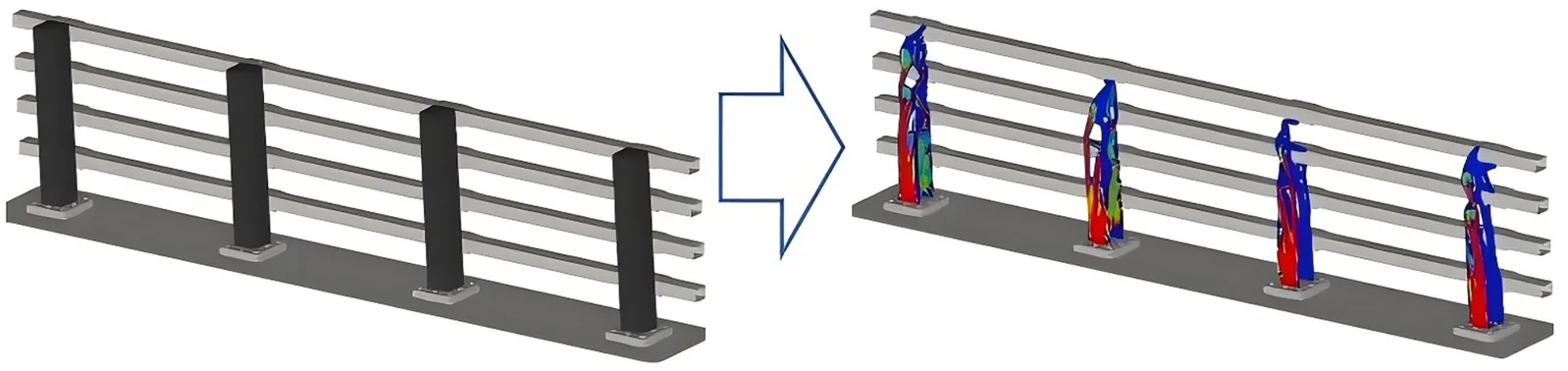
Topology optimization density contour of the posts.

**Fig 2:**
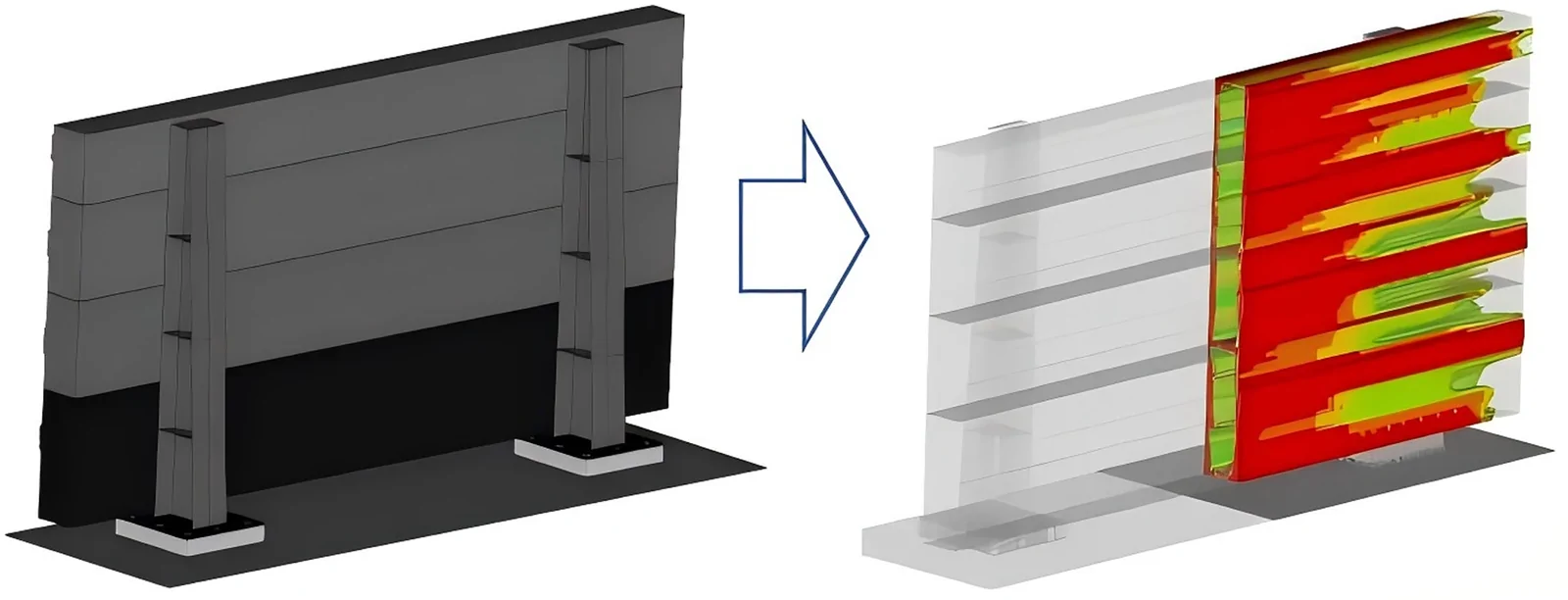
Topology optimization density contour of the beam.

The optimal distribution of the relative density (ρe) of materials in the design domain was obtained by solving the optimization model based on the SIMP method, as shown in Fig 1 (post) and Fig 2 (crossbeam). The contour map characterizes the priority of material distribution with a continuous color scale ranging from blue to red: the red area (ρe ≥ 0.8) identifies the main load transmission path where material is most needed, and the blue area (ρe ≤ 0.3) corresponds to the removable inefficient material that can be eliminated for lightweighting. Intermediate colors (yellow, green) represent transitional material densities. These density contours are direct outputs from the topology optimization solver, representing the optimized element relative density distribution.

(1) Material distribution of posts: High-density materials (ρe > 0.7) gather in the front collision surface, rear collision surface flange of the post and the root area close to the fixed constraint, forming a clear “double-limb” main force transmission skeleton. It indicates that under collision load, the post mainly resists the load through axial tension and compression of the front and rear flanges and bending at the root. The material density in the middle web area is low (0.3 < ρe < 0.6), which can be used as a secondary load-bearing or connecting component for lightweight treatment.
(2) Material distribution of crossbeams: The material is relatively uniformly distributed on the outer contour of the crossbeam section, especially forming a continuous high-density band (ρe > 0.8) at the upper and lower flanges. The crossbeam mainly bears bending load in the overall stress, and its optimal configuration should be a closed thin-walled component with high section moment of inertia.

**Fig 3:**
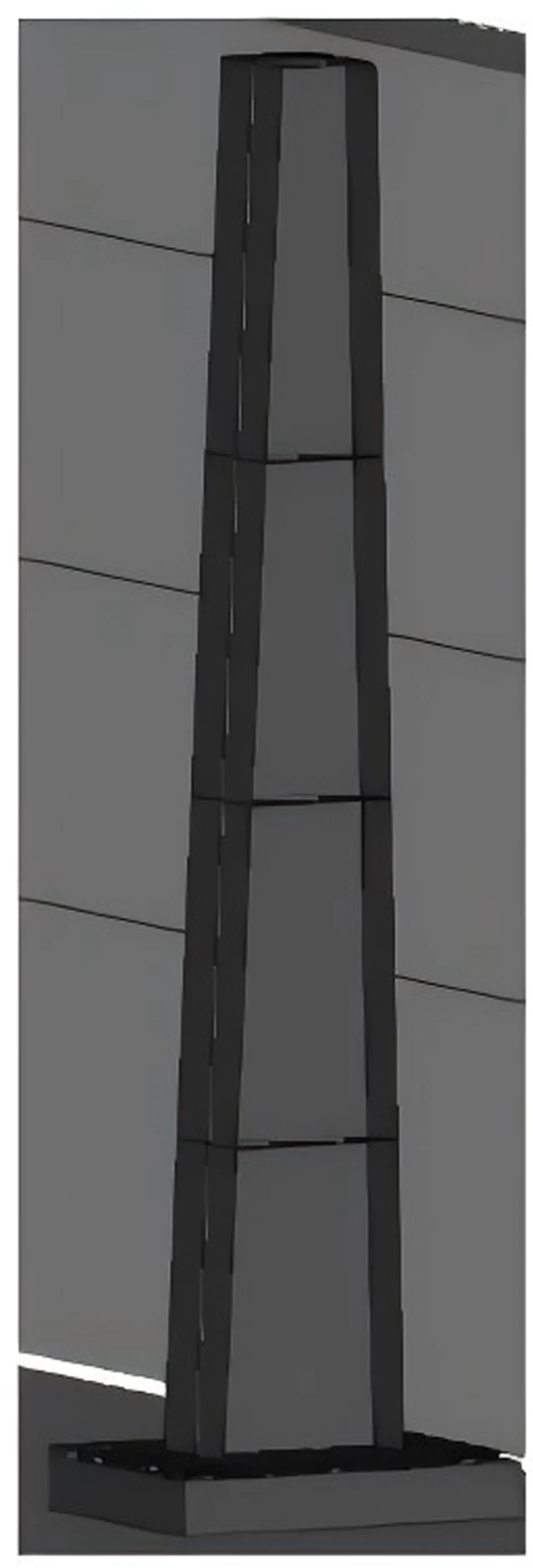
Inclined H-section post (based on contour evolution).

**Fig 4:**
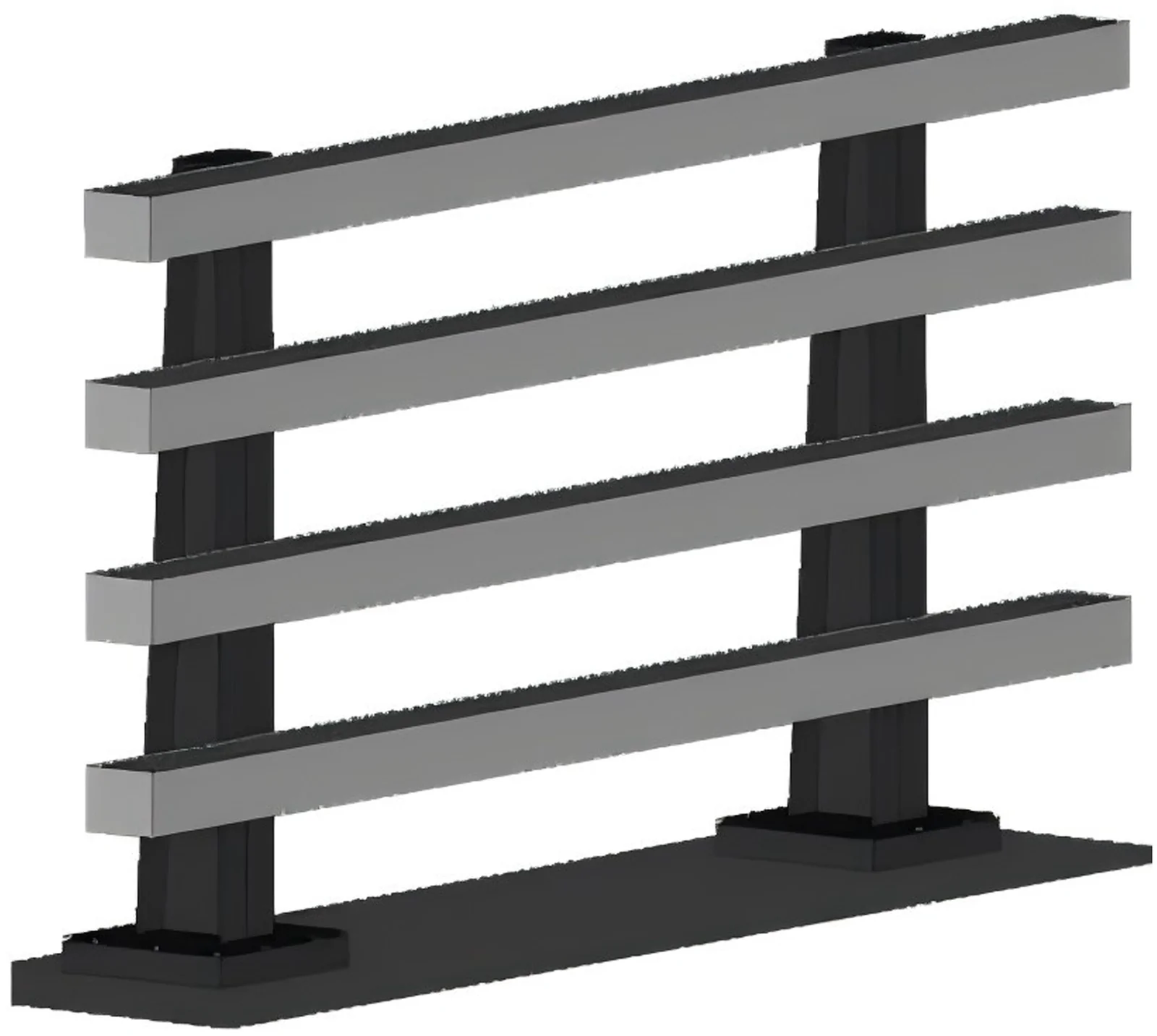
Rectangular section beam (based on contour evolution).

To make the topology optimization results engineering feasible and take into account the manufacturing process and connection requirements, the above configuration is evolved as follows:

1) Post configuration evolution: Inclined H-shaped section. According to the main material force transmission path, the post is specifically transformed into an inclined H-shaped section with a narrow top and a wide bottom (Fig 3). This evolution fully responds to the topology results: the wide bottom flange plate width is 0.26 m, corresponding to the high-density area, providing strong bending and shear resistance; the narrowed top flange plate width is 0.11 m, adapting to the material density gradient in the middle and upper parts. The overall post section dimensions are 0.5 m wide at the base (including the baseplate connection) tapering to 0.276 m at the top, with flange thicknesses of 8 mm. This forms a gradient stiffness distribution of “rigid bottom and flexible top.” This design uses the relatively flexible upper part to guide the vehicle to turn smoothly at the initial stage of collision, effectively reducing the collision force arm and inhibiting the vehicle rollover tendency in principle. The configuration is welded by flat plates with mature technology, ensuring good engineering economy.
2) Crossbeam configuration selection: Rectangular section tube. The topology results support the use of thin-walled closed sections as the optimal form of crossbeams. Under the constraint of the same steel consumption, the bending moment of inertia and anti-local crushing performance of circular, square and rectangular sections are compared. Mechanical analysis shows that the rectangular section tube (160 mm × 120 mm) has the highest section efficiency under the vertical bending condition dominated by vehicle collision (Fig 4). In addition, its flat and continuous front collision surface can evenly disperse the collision load, provide smoother guiding function, and significantly reduce the risk of vehicle tripping or loss of control due to local stress concentration.
(3) Final collaborative optimization structure: By deeply integrating the “on-demand material distribution” principle revealed by topology optimization with engineering practice, a new guardrail stress system characterized by “inclined H-shaped posts” and “rectangular tube crossbeams” is determined. This system not only realizes efficient material utilization in configuration, but also forms an organic collaboration between stiffness gradient and force flow path in mechanical mechanism, laying a structural foundation for subsequent anti-rollover performance and lightweight goals.

The topology optimization density contour data are available in S1 File.

### 2.3. Lightweight effectiveness

The weight per linear meter of the new HA-level guardrail based on 700L high-strength steel and topology optimization configuration is 180 kg. Taking the traditional Q355 steel HA-level guardrail commonly used in engineering (about 230 kg/m) as the benchmark, the weight is reduced by 50 kg/m, and the weight reduction rate reaches 21.7%. This effect comes from two major technical paths: first, the “high-strength thinning” effect of 700L high-strength steel, which greatly reduces the wall thickness of main components such as posts and crossbeams (for example, the thickness of the main post plate is reduced from 10 ~ 12 mm of the traditional one to 6 ~ 8 mm); second, the precise removal of materials in non-stressed areas by topology optimization to achieve “on-demand material distribution.” This quantitative result fully verifies the effectiveness of the design method in structural lightweighting.

## 3. Optimized guardrail structure scheme

**Fig 5:**
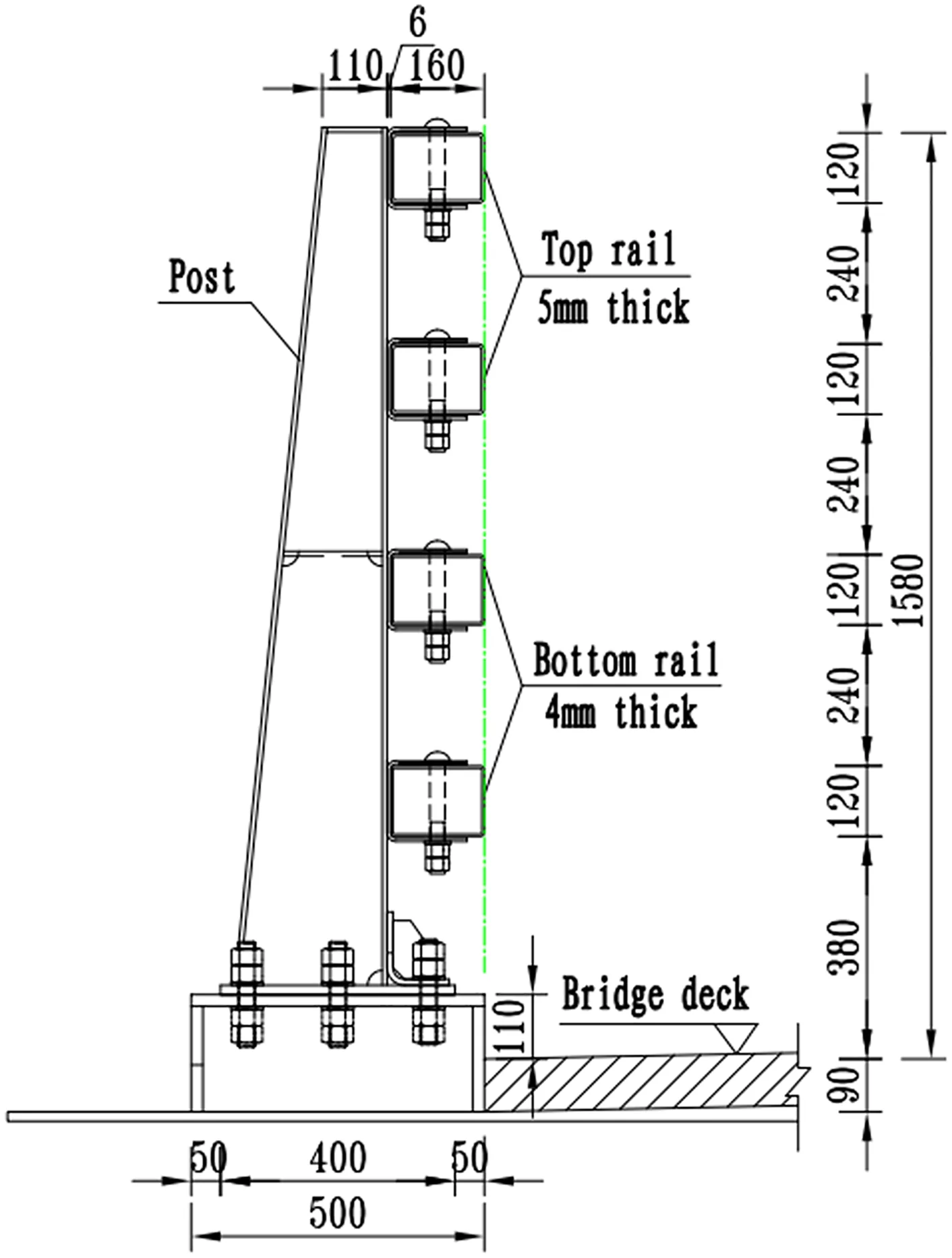
HA-level beam-post guardrail structure with all key dimensions annotated.

Based on the topological optimization results and configuration evolution from Section 2.2, the finalized design scheme for the HA-level beam-post guardrail primarily consists of an upper beam, a lower beam, posts, and a steel box-girder foundation, as shown in Fig 5. All key dimensions are annotated in Fig 5.

Inclined H-shaped post: Adopting 700L high-strength steel, the section is H-shaped with a narrow top and a wide bottom. Its main structural parameters are as follows: total post height 1.58 m; H-section bottom flange width 0.26 m; H-section top flange width 0.11 m; front and rear flange thickness 8 mm; web thickness 6 mm; baseplate 0.4 m wide × 0.02 m thick, welded to the post base; overall total width at the base including the baseplate connection 0.5 m; overall total width at the top of the H-section 0.276 m. This gives a gradient stiffness distribution of “rigid bottom and flexible top.”

Rectangular tube crossbeam: Adopting 700L high-strength steel, the thin-walled section size is 160 mm × 120 mm, the thickness of the upper two crossbeams is 5 mm, and the thickness of the lower two crossbeams is 4 mm.

Connection method: The crossbeam is connected to the post through Grade 10.9 high-strength bolts (M20 size, with corresponding nuts and washers), and the post baseplate is connected to the embedded steel flange connecting plate on the bridge deck through eight Grade 10.9 M24 high-strength bolts to form a collaborative stress whole. The cantilevered bridge deck for this test installation was a 0.25 m thick reinforced concrete deck with a 20 mm steel bearing plate embedded at each post location, anchored to the bridge box girder through shear studs. In permanent bridge installations, post spacing is typically 2.0 m on center.

## 4. Anti-rollover mechanism and full-scale crash test evaluation

### 4.1. Anti-rollover mechanism analysis

The vehicle rollover phenomenon during a collision with a guardrail fundamentally arises because the lateral impact force F acts below the vehicle's center of mass, generating a rolling moment M (M = F × h, where h is the vertical distance from the force application point to the vehicle's center of mass) that causes the vehicle to rotate about its longitudinal axis. Dangerous rollover occurs when this moment exceeds the vehicle's inherent stabilizing moment. It should be noted that the following analysis proposes a physical mechanism by which the gradient-stiffness post is expected to reduce the overturning moment; it is presented as a design rationale, whereas the full-scale tests in Section 4.2 report the directly measured containment, deformation, and observed vehicle attitude rather than direct roll-angle dynamics. The collaborative structure of “inclined H-shaped post-rectangular tube crossbeam” proposed in this paper inhibits this process from the root through the following three physical mechanisms:

(1) Reduce the overturning force arm and directly reduce the rolling moment. The gradient stiffness system of “stiff lower part and flexible upper part” formed by inclined H-shaped posts is critical. At the initial stage of collision, the vehicle first contacts the relatively flexible upper part of the post, causing controllable deformation, which effectively reduces the instantaneous action height h of the resultant collision force. According to M = F × h, for a given collision force F, decreasing the moment arm h directly reduces the roll moment M. This mechanism effectively weakens the initial roll tendency of the vehicle and avoids the lever effect caused by excessive upper stiffness in traditional uniform-section posts.
(2) Form a wide-area couple to enhance the anti-rollover countermoment of the system. The collaborative stress mode guided by topology optimization enables the single-point collision force to be quickly dispersed to adjacent multiple posts through the rectangular tube crossbeam with excellent continuity. At this time, multiple posts work together to form a resistance couple with a wider couple arm (approximately equal to the post spacing B) to balance the vehicle rolling moment M. The resistance couple moment is proportional to the width B of the couple arm, and the anti-rollover efficiency is significantly better than the traditional mode relying on single-point bending resistance. This mechanism is reflected in the improvement of the overall lateral stiffness of the guardrail system, which corresponds to a substantial reduction in the maximum lateral dynamic outward displacement W of the guardrail in the test results.
(3) Homogenize the contact load and smooth the instantaneous impact of the rolling moment. The rectangular tube crossbeam provides a continuous and smooth guiding plane, transforming the contact between the vehicle and the guardrail from the intermittent “point-line” contact in the traditional design to the large-area “surface-surface” contact. This design averages the concentrated collision impact force into a continuous pressure distribution, avoiding the instantaneous severe rolling moment caused by local stress peaks. The smooth guiding and load transmission process provides sufficient response and adjustment time for the vehicle suspension system, effectively maintaining the vehicle's attitude stability and preventing dynamic instability caused by instantaneous moment impact.

### 4.2. Full-scale vehicle crash test verification

#### 4.2.1. Test setup.

**Fig 6:**
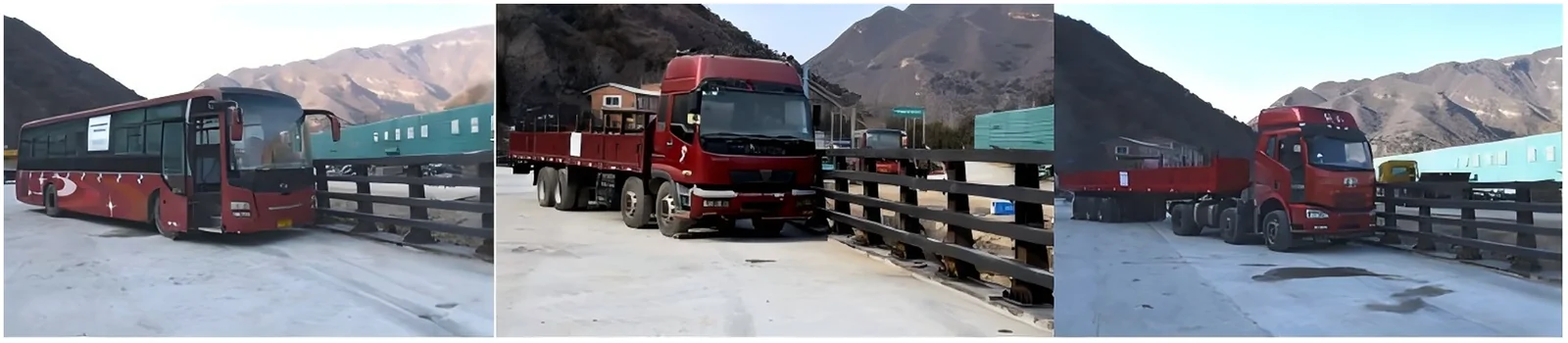
Full-scale vehicle crash tests for three test vehicles, including a 25 t extra-large bus, a 40 t large truck, and a 55 t tractor-semitrailer.

In accordance with the “Technical Standard for Safety Performance Evaluation of Highway Guardrails” (JTG B05-01–2013, Clause 6.2.2 for HA-level test conditions), full-scale vehicle crash tests were carried out on the optimized HA-level beam-post steel guardrail. The test matrix (Table 2) exactly matches the HA-level heavy vehicle conditions specified in the standard: a 25 t extra-large bus at 85 km/h (measured 86.0 km/h) at 20° (measured 20.3°), a 40 t large truck at 65 km/h (measured 66.0 km/h) at 20° (measured 20.1°), and a 55 t tractor-semitrailer at 65 km/h (measured 65.9 km/h) at 20° (measured 20.2°). The acceptance criterion for the maximum dynamic equivalent vehicle lateral intrusion value, VIn ≤ 1.5 m, is specified in Clause 6.2.5 of the same standard. The test guardrail was installed through the steel box girder base, the installation height of the test section was 1.58 m, and the length was 42.00 m. In strict accordance with the HA-level heavy-vehicle test matrix, three typical heavy-duty vehicle models were selected for full-scale vehicle crash tests (Fig 6). The detailed test conditions are presented in Table 2.

**Table 2:** Conditions of full-scale vehicle crash test for HA-level guardrail.

Collision Vehicle Type	Total Vehicle Mass (kg)	Collision Speed (km/h)	Collision Angle (°)	Collision Energy (kJ)
Extra-large bus	25,132	86.0	20.3	863
Large truck	40,158	66.0	20.1	797
Tractor-semitrailer	55,144	65.9	20.2	1101

#### 4.2.2. Test results.

**Fig 7:**
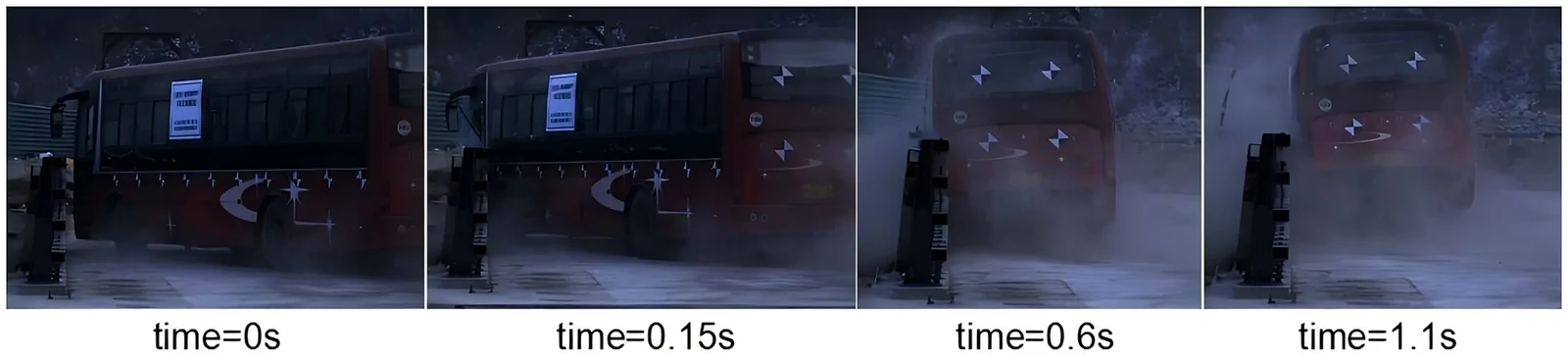
Post-impact attitude of extra-large bus. Note: The original photographs are affected by dust and debris; annotated key frames are provided in S1 Fig to facilitate assessment of vehicle attitude.

**Fig 8:**
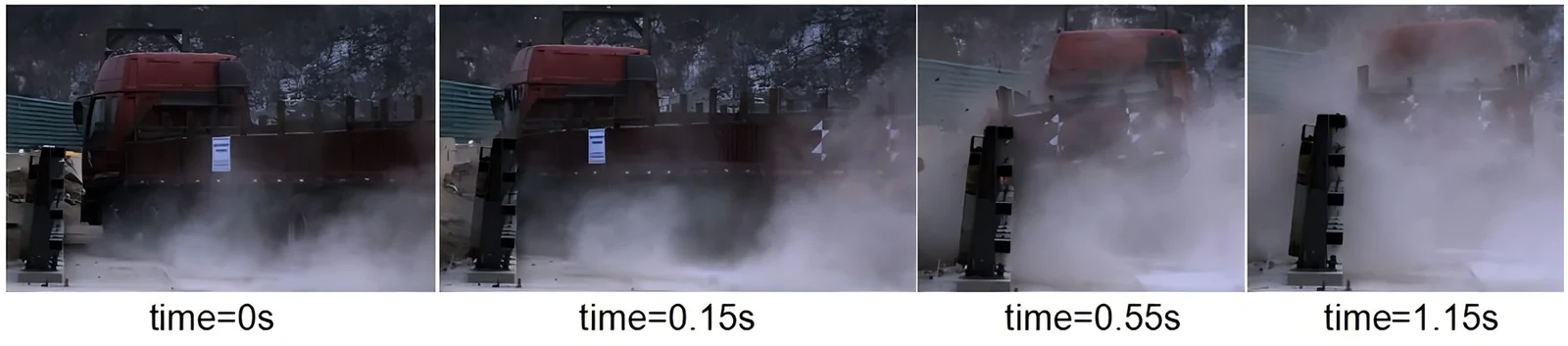
Post-impact attitude of large truck. Note: The original photographs are affected by dust and debris; annotated key frames are provided in S2 Fig to facilitate assessment of vehicle attitude.

**Fig 9:**
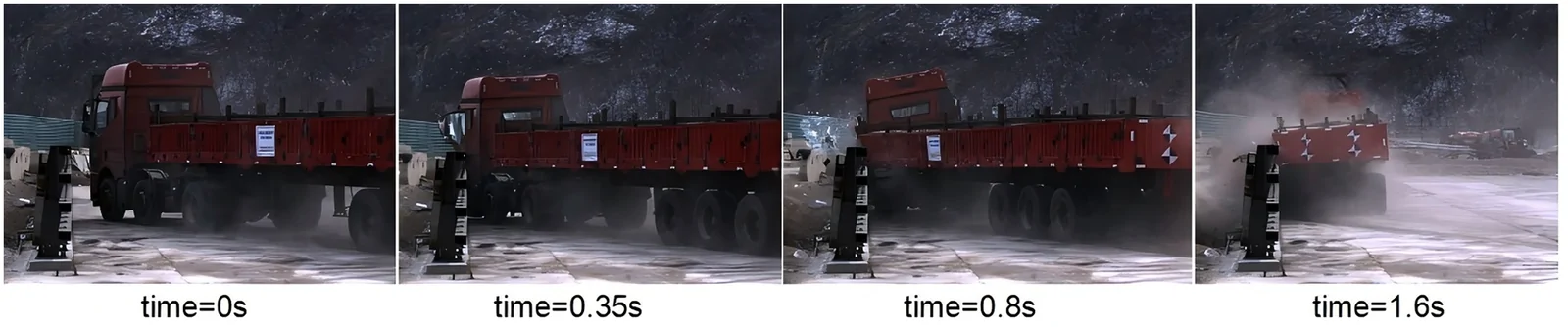
Post-impact attitude of 55 t tractor-semitrailer. Note: The original photographs are affected by dust and debris; annotated key frames are provided in S3 Fig to facilitate assessment of vehicle attitude.

The test results are shown in Figs 7–9. It should be noted that the dust and debris visible in these photographs are inherent to full-scale heavy vehicle crash testing on concrete bridge decks; the high-speed video footage (recorded at 500 fps from multiple angles) provided clearer observation of vehicle attitude throughout the impact event. Under the impact conditions of the three vehicle types, the optimized guardrail effectively prevented vehicle penetration, override, and straddling. No guardrail components or detached parts intruded into the passenger compartment. All vehicles maintained a stable post-crash attitude and met the requirements for guided exit. Post-test inspection revealed controlled plastic deformation of the impacted posts and beams, with minor localized spalling of the concrete deck surface at the immediate impact location; no structural damage to the deck anchorage or embedded steel plates was observed, and no bolt failures occurred.

Because the original photographs in Figs 7–9 are affected by dust and debris, annotated key frames extracted from the 500-fps high-speed video are provided as S1–S3 Figs. These frames show the vehicle at pre-impact, first contact, maximum lateral displacement, and exit, with the vehicle longitudinal axis and the guardrail reference line overlaid. Visual inspection of these frames indicates that no wheel lift or substantial body roll was observed in any test; all vehicles remained substantially upright throughout the impact sequence and exited with stable attitude.

For clarity, the key displacement metrics are defined as follows: D = maximum lateral dynamic deformation of the guardrail itself (permanent plastic deformation at the most impacted post); W = maximum lateral dynamic extension of the guardrail system including the displaced vehicle contact point (i.e., how far the guardrail-vehicle interface moves laterally); VI = maximum dynamic lateral displacement of the vehicle body outer edge relative to its original path; VIn = maximum dynamic equivalent vehicle lateral intrusion value, which is the normalized composite metric used in JTG B05-01–2013 combining guardrail deformation and vehicle lateral displacement, representing the total lateral intrusion of the vehicle-guardrail system into the protected area behind the guardrail.

A quantitative comparison between the optimized guardrail and the conventional guardrail [20] was conducted under three standard HA-level heavy-vehicle crash conditions. Note that the conventional guardrail reference data from Pei et al. [20] (co-authored by corresponding author S. Gong) was obtained under identical test conditions (same vehicle types, speeds, angles, and acceptance criteria per JTG B05-01–2013) at the same test facility, ensuring a like-for-like comparison. The results are summarized in Table 3. It should also be noted that the conventional guardrail's maximum VIn of 1.40 m already satisfies the ≤ 1.5 m HA-level acceptance criterion; the improvement reported here represents a substantial margin enhancement rather than a fail-to-pass transition.

**Table 3:** Summary of key results of full-scale vehicle crash test for HA-level guardrail.

Evaluation Item	Optimized guardrail			Conventional guardrail [20]		
	Extra-large Bus	Large Truck	Tractor-semitrailer	Extra-large Bus	Large Truck	Tractor-semitrailer
Maximum lateral dynamic deformation D of guardrail (m)	0.15	0.15	0.10	0.30	0.35	0.55
Maximum lateral dynamic extension W of guardrail (m)	0.35	0.35	0.30	0.70	0.70	0.95
Maximum dynamic lateral displacement of vehicle, VI (m)	0.35	0.25	0.25	0.65	0.65	1.10
Maximum dynamic equivalent vehicle lateral intrusion value VIn (m)	0.45	0.50	0.50	0.80	0.80	1.40

Note: W denotes the maximum lateral extension of the guardrail-vehicle contact interface during impact; the conventional guardrail design details are documented in Pei et al. [20]. In Chinese bridge engineering practice, these systems are referred to as “guardrails” rather than “bridge rails.”

The optimized guardrail exhibited significantly reduced lateral intrusion under all tested conditions. Compared with the conventional guardrail, the maximum dynamic equivalent vehicle lateral intrusion value (VIn) was reduced by 43.8%, 37.5%, and 64.2% for the extra-large coach, heavy truck, and tractor-semitrailer crash conditions, respectively. This indicates that the collaborative structure composed of inclined H-shaped posts and rectangular tube beams possesses good adaptability and stability under impacts with different masses and collision energies. Under the 55 t tractor-semitrailer condition (1101 kJ), which involves the highest collision energy, the VIn value of the optimized guardrail was reduced from 1.40 m to 0.50 m, representing a reduction of 64.2%.

The test results are consistent with the anti-rollover mechanism proposed in Section 4.1. During the collision, the stable contact and controllable attitude change of the vehicle are consistent with the expected effect of the “stiff lower part and flexible upper part” post configuration in reducing the initial overturning moment arm. The small and uniform overall lateral deformation (values D and W) of the guardrail indicates that the impact force was distributed through the beam to multiple posts, forming a wide-range resisting couple. The substantial reduction in vehicle lateral displacement (VI and VIn) shows that this structural system was effective in homogenizing loads and mitigating moment impact. Multi-angle 500-fps high-speed video recordings confirmed that all three vehicles maintained stable post-impact attitudes without rollover, override, straddling, or any observable tendency toward excessive rolling, pitching, or yawing instability.

It should be clarified that the quantitative metrics reported above (D, W, VI, and VIn) characterize guardrail deformation and vehicle lateral intrusion, which are the standard acceptance quantities in JTG B05-01–2013; direct rollover variables—roll angle, lateral acceleration, and yaw rate—were not instrumented in this test series. Accordingly, anti-rollover performance is assessed in this study at two levels: (i) the binary outcome required by the standard, namely that none of the three vehicles rolled over, overrode, or straddled the guardrail and that all exited with stable attitude, as determined from the multi-angle 500-fps high-speed video; and (ii) indirect support from the substantially reduced lateral intrusion, which indicates suppression of the vehicle climbing/override precursor to rollover. The moment-arm mechanism proposed in Section 4.1 is consistent with these observations but is not treated as a direct quantitative verification of rollover resistance. Onboard IMU instrumentation to measure roll angle, lateral acceleration, and yaw rate time histories is planned for the next test campaign. Regarding occupant risk indices, while the Chinese standard JTG B05-01–2013 emphasizes vehicle containment and trajectory metrics for heavy-vehicle HA-level tests rather than the ASI/THIV indices used in EN 1317 for passenger car tests, visual observation confirmed no cabin intrusion and the vehicle deceleration profiles appeared qualitatively consistent with acceptable occupant risk levels. The collaborative design proposed in this paper optimizes the vehicle-guardrail collision interaction from the mechanical mechanism and achieves an improvement in vehicle attitude stability and lateral intrusion resistance.

## 5. Discussion

Building upon the full-scale crash test results presented in Section 4, this section provides a comprehensive discussion of the findings. It delves into the synergistic benefits of the proposed material-structure collaborative design, elucidates the physical anti-rollover mechanisms, and benchmarks the new guardrail's performance against existing technologies. Furthermore, it addresses the study's limitations and outlines future research directions, thereby contextualizing the significance of this work within the broader field of bridge engineering.

### 5.1. Synergistic benefits of material-structure collaborative design

The integration of 700L high-strength steel with topology optimization represents a paradigm shift from conventional empirical design methodologies. The measured yield strength of 700L (626–720MPa) is approximately double that of the Q355 standard minimum (Table 1), facilitating a direct thickness reduction of 30–40% in primary load-bearing components. However, the principal innovation extends beyond mere material substitution; it lies in the systematic synthesis of advanced material properties with structurally optimized configurations.

The topology optimization results (Fig 1) revealed that an inclined H-shaped post exhibiting a gradient stiffness distribution (rigid at the base, flexible at the top) constitutes the optimal load transfer path under collision scenarios. This finding challenges the conventional wisdom of uniform-section posts and provides a mechanistic explanation for the observed 64.2% reduction in vehicle dynamic lateral displacement (VIn) under the most severe 55 t tractor-semitrailer impact. This gradient stiffness configuration is expected to diminish the initial overturning moment arm by permitting controlled deformation of the upper post section, thereby mitigating the rolling moment that can lead to vehicle rollover.

Furthermore, the rectangular tube crossbeam (160 mm × 120 mm) identified through topology optimization offers superior sectional efficiency compared to circular or square alternatives. Its continuous frontal surface transforms vehicle-guardrail interaction from intermittent point-line contact to distributed surface-surface contact, homogenizing impact loads and facilitating smoother vehicle redirection. This observation aligns with recent studies emphasizing the critical role of contact surface continuity in crashworthiness performance [13,14,21]. Collectively, these synergistic mechanisms establish that the proposed methodology yields an HA-level guardrail achieving simultaneous improvements in safety performance, lightweighting (21.7% weight reduction), and economic efficiency (10–15% material cost reduction), thereby addressing the critical challenge of balancing safety, weight, and cost in high-performance bridge guardrails.

Despite these advantages, it is important to acknowledge the fabrication considerations associated with 700L high-strength steel. Its higher carbon equivalent necessitates careful control of welding parameters (e.g., pre-heating, interpass temperature, and the use of low-hydrogen welding consumables) to prevent cold cracking. However, established welding procedures are well-documented for such steels and have been successfully implemented in heavy machinery and automotive industries. Furthermore, the material cost of 700L is approximately 15–20% higher than Q355, but this is offset by the overall 21.7% weight reduction, leading to a net decrease in total material cost as detailed in the economic analysis. Regarding the concern about low-temperature brittleness, while the ductile-to-brittle transition temperature is a valid consideration for any BCC steel, the specified minimum elongation (≥20.5%) of the 700L used in this study indicates sufficient ductility. Future validation under extreme low-temperature conditions, as noted in Section 5.5, will provide a definitive safety margin for cold-region applications.

### 5.2. Anti-rollover mechanism: from empirical observation to physical principle

While previous research has documented the rollover phenomenon during guardrail collisions [7–10], few studies have systematically elucidated the underlying physical mechanisms or proposed design strategies specifically targeting rollover mitigation. This study contributes a physically grounded framework for understanding and suppressing vehicle rollover through three interconnected mechanisms, as initially proposed in Section 4.1:

(1) Moment arm reduction via gradient stiffness distribution;
(2) Formation of wide-area resisting couples through multi-post collaboration;
(3) Load homogenization via continuous contact surfaces.

The full-scale crash test results provide validation of this framework. Under the 55 t tractor-semitrailer condition, the maximum guardrail lateral dynamic deformation (D) was limited to 0.10 m, compared to 0.55 m for conventional guardrails [20]. This 81.8% reduction in deformation directly confirms the effectiveness of the multi-post collaborative resistance mechanism, wherein impact forces are rapidly distributed through the continuous rectangular beams to adjacent posts, creating a resisting couple with an effective moment arm approximately equal to the post spacing.

Importantly, the proposed mechanisms operate synergistically rather than independently. The initial moment arm reduction facilitated by the flexible upper post section attenuates the rolling moment magnitude, while the wide-area resisting couple provides enhanced countermoment capacity. Concurrently, load homogenization prevents instantaneous moment spikes that could destabilize the vehicle suspension system. This multifaceted approach represents a significant advancement over conventional designs that rely primarily on single-point bending resistance.

### 5.3. Lightweighting implications for bridge engineering

The 21.7% reduction in weight per linear meter (from 230 kg/m to 180 kg/m) achieved in this study carries substantial implications for long-span cable-supported bridges, where dead load constitutes a critical design consideration. For a typical cable-stayed bridge with 2 km of guardrail on each side, the total weight reduction amounts to approximately 200 tonnes. This reduction translates directly into decreased material consumption in primary load-bearing components such as stay cables, pylons, and foundations, potentially yielding cascading cost savings.

Economic analysis reveals that despite the higher unit cost of 700L high-strength steel (approximately 15–20% above Q355), an overall material cost reduction of 10–15% is achievable due to decreased steel consumption. When considering indirect benefits—including reduced transportation and installation costs, diminished foundation requirements, and extended service life due to enhanced corrosion resistance of high-strength low-alloy steels—the comprehensive techno-economic viability becomes even more compelling. These findings address a critical gap in the literature, where economic assessments of high-performance guardrails have been notably absent [5,6].

### 5.4. Comparison with existing guardrail technologies

To further contextualize the performance of the proposed guardrail, Table 4 presents a comparative analysis with representative existing technologies reported in the literature.

**Table 4:** Comparative performance of guardrail systems.

Guardrail Type	Material	Weight (kg/m)	Max VI_n_(m)	Protection Level	Reference
Concrete barrier	C50	650	0.30	HA	[4]
Conventional beam-post	Q355	230	1.40	HA	[20]
This study	700L	180	0.50	HA (heavy-vehicle conditions)	—

It should be noted that while the concrete barrier shows a lower VIn, its excessive self-weight (650 kg/m) poses significant challenges for long-span bridge applications. Compared with the conventional steel guardrail of the same HA level, the proposed design achieves a 21.7% weight reduction and a 64.2% reduction in maximum VIn (lateral intrusion).

The proposed guardrail achieves the lowest weight among HA-level steel systems while maintaining superior lateral intrusion resistance. Compared to concrete barriers, which offer excellent vehicle containment but suffer from excessive self-weight and poor permeability, the proposed steel guardrail provides a balanced solution particularly suitable for long-span bridges where dead load reduction is paramount. The 0.50 m maximum VIn value comfortably satisfies the HA-level requirement (≤1.5 m) with a substantial safety margin, and approaches the performance of rigid concrete systems while offering the advantages of steel construction, including prefabrication, modular installation, and enhanced aesthetic integration with bridge superstructures.

Note: For the proposed guardrail, HA-level protection is established for the tested heavy-vehicle conditions; the passenger car test required for full HA-level certification is planned but not yet completed.

### 5.5. Study limitations and future research directions

Despite the promising results, several limitations warrant acknowledgment and provide directions for future research:

(1) Impact condition generalizability: While this study evaluated three representative heavy vehicle types under standard HA-level conditions, real-world collision scenarios involve greater variability in vehicle types, impact angles, speeds, and environmental conditions. Future studies should investigate guardrail performance under a broader range of impact conditions, including passenger vehicles which constitute the majority of traffic volume. Specifically, the HA-level evaluation standard also includes a 1.5 t passenger car collision test at 100 km/h and a 20° angle. Given that this condition involves a significantly lower center of mass and collision energy compared to the heavy vehicles tested here, the interaction with the guardrail's lower beam section and the consequent vehicle stability mechanism are distinct and warrant dedicated investigation. Although the standard's heavy-vehicle conditions are generally more critical for evaluating the guardrail's strength and anti-rollover capacity, a full evaluation including the passenger car condition is necessary for a comprehensive safety assessment and is planned for the next phase of this research.
(2) Long-term durability: The corrosion performance of 700L high-strength steel in bridge environments, particularly under de-icing salt exposure, requires systematic investigation. Although high-strength low-alloy steels generally exhibit enhanced atmospheric corrosion resistance compared to conventional carbon steels [22], long-term field monitoring and accelerated laboratory testing are necessary to quantify service life and establish maintenance protocols.
(3) Multi-objective optimization refinement: The current topology optimization employed static load simplification based on energy equivalence principles. Future work should incorporate explicit dynamic finite element analysis (e.g., LS-DYNA) within the optimization framework to capture strain-rate effects, material nonlinearity, and large deformation behavior. A single validating dynamic FE run of the as-built H-post/rectangular-tube guardrail under the 55 t tractor-semitrailer crash condition would provide a numerical link between the optimization results and the physical test, and is planned for subsequent publication. Advanced optimization algorithms, such as evolutionary structural optimization or level-set methods, may yield further configuration improvements. In addition, onboard IMU and GPS units will be installed in the next test campaign to directly measure roll angle, lateral acceleration, and yaw rate time histories, providing quantitative rollover dynamics that complement the video-based attitude assessment and intrusion metrics reported here.
(4) Connection detailing and fatigue performance: The bolted connections between crossbeams and posts, while practical for construction, represent potential fatigue-sensitive details under repeated service loads. Although guardrails are primarily designed for ultimate limit states (collision events), fatigue performance under traffic-induced vibrations warrants investigation, particularly for bridge applications.
(5) Full-scale validation across temperature ranges: The current crash tests were conducted under ambient conditions. Given the temperature sensitivity of steel fracture toughness, particularly for high-strength grades, additional validation at low temperatures (e.g., −30°C to −40°C) is recommended for bridges in cold regions to ensure ductile behavior.

### 5.6. Broader implications for structural engineering practice

Beyond the specific application to bridge guardrails, this study demonstrates the transformative potential of integrating advanced materials with computational design optimization in structural engineering. The methodological framework—comprising material characterization, topology optimization, configuration evolution, full-scale validation, and economic assessment—provides a template for lightweight design of other safety-critical infrastructure components.

The successful translation of abstract topology optimization results into manufacturable, constructible configurations through engineering interpretation represents a critical contribution. While topology optimization has been extensively applied in aerospace and automotive industries, its adoption in civil infrastructure has been limited by challenges related to scale, manufacturing constraints, and regulatory compliance. This study demonstrates that with appropriate interpretation and configuration evolution, topology-optimized designs can satisfy both performance requirements and practical construction considerations, thereby paving the way for broader adoption of computational design methods in structural engineering.

## 6. Conclusions

By combining the material advantages of 700L high-strength alloy steel with the structural design of topology optimization, a new type of lightweight extra-high grade (HA-level) beam-post guardrail is proposed and verified through full-scale crash testing. The main conclusions are as follows:

(1) The adoption of 700L high-strength steel (yield strength ≥626 MPa, elongation after fracture ≥20.5%) not only provides the material basis for “high-strength and thinning,” reducing the guardrail weight per meter by 21.7%, but its enhanced strength-ductility product (14.4–21.4 GPa·%) also ensures high energy absorption capacity under extreme collision conditions.
(2) Based on topology optimization, a synergistic structure composed of “inclined H-shaped posts” and “rectangular tube beams” was obtained, achieving a gradient stiffness distribution characterized as “stiff-bottom, flexible-top” and efficient material utilization. This advances guardrail design from traditional empirical models towards performance-based, digital, and integrated design.
(3) Full-scale vehicle crash tests covering the three HA-level heavy-vehicle conditions specified in JTG B05-01—2013—25 t bus, 40 t truck, and 55 t tractor-semitrailer—confirm that the guardrail meets the containment and redirection requirements for those heavy-vehicle conditions. None of the three vehicles rolled over, overrode, or straddled the guardrail, and all exited with stable attitude. The passenger car condition specified in the same HA-level test matrix was not included in the present study and remains to be evaluated separately. Under the 55 t tractor-semitrailer collision condition, the maximum dynamic equivalent vehicle lateral intrusion value (VIn) is reduced by 64.2% compared with the conventional guardrail. These observations are consistent with the proposed mechanism of suppressing the roll moment by reducing the collision force arm and forming a wide-range resisting couple, although direct roll-angle measurement is needed to quantitatively characterize rollover resistance.
(4) Although the unit price of 700L steel is higher, the 21.7% weight reduction results in an estimated 10–15% direct material cost saving. A preliminary life-cycle cost assessment, factoring in reduced transportation, installation, and bridge dead load, indicates significant economic advantages over conventional HA-level steel guardrails, warranting a full project-specific cost-benefit analysis.

## Supporting information

S1 File Topology optimization density contour data. (ZIP)

S1 Fig Annotated high-speed video key frames for the 25 t bus crash test. (ZIP)

S2 Fig Annotated high-speed video key frames for the 40 t truck crash test. (ZIP)

S3 Fig Annotated high-speed video key frames for the 55 t tractor-semitrailer crash test. (ZIP)
